# Research progress on the BAG3-HSP70 axis-mediated autophagy in acute myeloid leukemia

**DOI:** 10.3389/fcell.2026.1980188

**Published:** 2026-10-14

**Authors:** Yang Liu, Tao Wu

**Affiliations:** Department of Hematology, People’s Liberation Army Joint Logistics Support Force 940th Hospital, Lanzhou, China

**Keywords:** acute myeloid leukemia, autophagy, BAG3, chaperone-assisted selective autophagy, chemoresistance, HSP70, leukemia stem cells, targeted therapy

## Abstract

Acute myeloid leukemia (AML) is a genetically heterogeneous hematological malignancy in which chemoresistance and relapse, often driven by leukemia stem cells (LSCs), remain major obstacles to cure. Autophagy is an evolutionarily conserved lysosomal degradation pathway whose function in AML is not fixed but is stage- and context-dependent: it restrains leukemogenesis in some settings while sustaining established and therapy-stressed LSCs in others. The Bcl-2-associated athanogene 3 (BAG3)-heat shock protein 70 (HSP70) axis integrates proteotoxic stress with the autophagy machinery, most notably through chaperone-assisted selective autophagy (CASA). This review applies a four-tier evidence framework—direct AML evidence, other hematological evidence, non-AML evidence, and proposed or hypothetical mechanisms—and explicitly separates BAG3-specific evidence from general HSP70 and autophagy findings. Direct AML evidence is comparatively strong for the cytoprotective, mitophagic and chemoresistance roles of autophagy and HSP70, but it is limited for a specific BAG3 dependency. In particular, direct BAG3 coupling to ULK1, competitive BAG3/HSP70 binding at the Beclin1 BH3 domain, and direct HSP70 shielding of ATG5 from cleavage are not supported by primary evidence. We examine the crosstalk between this axis and recurrent AML alterations (FLT3-ITD, NPM1 and TP53), and survey therapeutic strategies ranging from ATP-site HSP70 inhibitors to HSP70-BAG3 protein-protein interaction inhibitors, dual HSP70/HSP90 targeting, BAG3-directed agents, targeted protein degradation, nanoparticle delivery and emerging immunotherapies. We stratify direct axis-targeting from broad HSP70-family or proteostasis approaches. Finally, we summarize the principal knowledge gaps and the potential therapeutic window, cardiac-safety and biomarker challenges that must be addressed to convert the BAG3-HSP70 axis from a plausible mechanism into a precision target in AML.

## 1 Introduction

Acute myeloid leukemia (AML) arises from the malignant transformation of hematopoietic stem or progenitor cells and is defined by the clonal expansion of immature myeloid blasts. Recurrent alterations such as FLT3-ITD, mutant NPM1 and TP53 mutations delineate molecular subtypes with markedly different outcomes (Döhner et al., 2022). Despite intensive chemotherapy, hypomethylating agents, venetoclax-based regimens and targeted inhibitors, long-term survival remains unsatisfactory, particularly in older and relapsed/refractory (R/R) patients, because minimal residual disease and leukemia stem cells (LSCs) survive treatment and drive relapse. Both the proteostasis network and autophagy have consequently emerged as candidate dependencies of the resistant leukemic compartment (Lam et al., 2026; Chen et al., 2024).

Autophagy is a highly conserved catabolic process in which cytoplasmic material is sequestered in double-membrane autophagosomes and delivered to lysosomes for degradation, supplying recycled nutrients and removing damaged organelles and misfolded proteins. Its role in cancer is dual and context dependent: it can suppress early tumorigenesis yet support established tumors under metabolic, hypoxic or therapeutic stress. In AML, autophagy is intertwined with metabolism, mitochondrial quality control and LSC maintenance, and is frequently mobilized as a protective response to targeted therapy (Saulle et al., 2023; Joffre et al., 2021; Ianniciello et al., 2018).

BAG3 is a stress-inducible co-chaperone and nucleotide exchange factor for HSP70 that, together with HSPB8, HSP70, CHIP and p62, nucleates the CASA machinery and also scaffolds several signaling complexes (Rosati et al., 2011; Tedesco et al., 2023a). BAG3 is upregulated in many tumors and has been implicated in the survival of normal and neoplastic leukocytes (Bonelli et al., 2004; Liu et al., 2022). A central caveat, however, is that the evidence in AML is considerably stronger for autophagy and HSP70 than for a specific BAG3-HSP70/CASA dependency. Direct AML evidence that BAG3 couples to the core ULK1 or Beclin1 initiation/nucleation machinery is still lacking, and several mechanistic claims have been extrapolated from solid-tumor or muscle models. Here we first summarize BAG3-HSP70 structure and define the distinctions between the principal autophagy pathways, then provide an inventory of the direct evidence for BAG3 in AML and critically evaluate, with explicit four-tier evidence grading and a careful separation of autophagy induction from flux, the proposed roles of the axis at each autophagy stage and its stage-dependent duality (Figure 1), its crosstalk with FLT3-ITD, NPM1 and TP53, and finally the expanding therapeutic landscape, including protein-protein interaction and dual-chaperone targeting, degradation and delivery technologies, and emerging immune-based approaches.

**FIGURE 1:**
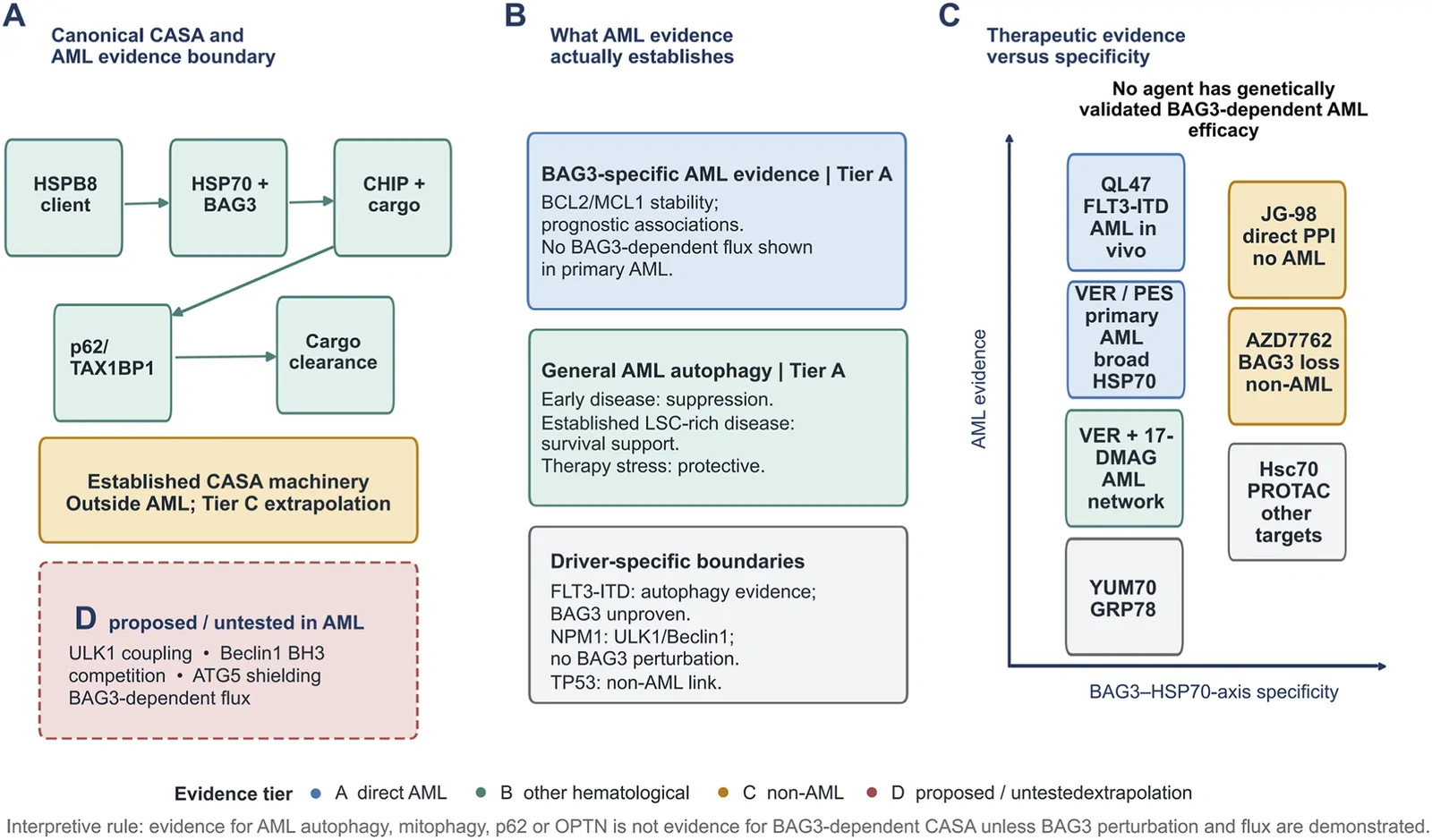
Evidence map for the BAG3–HSP70 axis, autophagy and therapeutic targeting in AML. **(A)** Canonical CASA proceeds through HSPB8-mediated client recognition, HSP70–BAG3 triage, CHIP-dependent ubiquitination, receptor/LC3 loading and lysosomal degradation. This machinery is established mainly outside AML (Tier C). Direct BAG3–ULK1 coupling, BAG3/HSP70 competition at the Beclin1 BH3 groove, HSP70 shielding of ATG5 from calpain cleavage and BAG3-dependent CASA flux have not been demonstrated in AML and are explicitly classified as Tier D (proposed or untested). Within panel A, the red dashed box labeled “D” summarizes the proposed/untested (Tier D) links—BAG3—ULK1 coupling, Beclin1 BH3 competition, ATG5 shielding and BAG3-dependent flux—and is an inset within A, not a fourth panel. **(B)** Direct AML data support BAG3-associated anti-apoptotic stability and prognostic or treatment-response associations, whereas the stronger functional evidence for autophagy concerns general autophagy and mitophagy. FLT3-ITD, NPM1 and TP53 evidence is therefore bounded separately. **(C)** Therapeutic approaches are positioned by direct AML evidence and specificity for the BAG3–HSP70 interaction. QL47, VER-155008, PES and dual HSP70/HSP90 inhibition have AML evidence but are broad chaperone or proteostasis interventions; JG-98 directly disrupts the HSP70–BAG3 interaction but has not been tested in AML. Hsc70-recruiting PROTACs use the chaperone machinery to degrade other target proteins and are not HSP70 degraders. CASA, chaperone-assisted selective autophagy; PPI, protein–protein interaction.

## 2 The BAG3-HSP70 axis: structure and canonical functions in autophagy

### 2.1 HSP70 family: molecular chaperones and pleiotropic autophagy regulators

The HSP70 family comprises highly conserved ATP-dependent molecular chaperones that include the constitutive HSC70 (HSPA8), the stress-inducible HSP70 (HSPA1A/HSPA1B), the mitochondrial HSPA9 and the endoplasmic reticulum (ER) paralog GRP78/HSPA5 (Radons, 2016). Each HSP70 protein contains an N-terminal nucleotide-binding domain (NBD), a flexible linker and a C-terminal substrate-binding domain (SBD) divided into a beta-sandwich and an alpha-helical lid. Cycling between ATP- and ADP-bound states, modulated by J-domain co-chaperones and nucleotide exchange factors, governs substrate capture and release and underlies *de novo* folding, refolding and trafficking (Balchin et al., 2020; Backe et al., 2025).

Beyond canonical folding, HSP70 is a pleiotropic regulator of proteostasis and autophagy: it participates in chaperone-mediated autophagy, in CASA cargo handling and in lysosomal and organelle quality control, and it is frequently upregulated by heat-shock factor 1 (HSF1) under proteotoxic stress. In AML, HSP70 is overexpressed in a substantial proportion of cases and is associated with adverse prognostic features, whereas elevated circulating HSP70 has been correlated with tumor burden and inferior survival (Thomas et al., 2005; Yeh et al., 2010). Recent systems-level work identifies the proteostasis network, including HSP70, as a therapeutic vulnerability in AML (Lam et al., 2026).

### 2.2 BAG3: co-chaperone and scaffold for autophagy signaling

BAG3 belongs to the Bcl-2-associated athanogene (BAG) family of nucleotide exchange factors, defined by a C-terminal BAG domain that binds the HSP70 NBD and accelerates ADP/ATP exchange, thereby biasing substrate handling (Rosati et al., 2011). In addition to the BAG domain, BAG3 contains an N-terminal WW domain, two PxxP motifs and an IPV region that binds the small heat shock protein HSPB8; these modules allow BAG3 to act as a multifunctional scaffold (Zamotina et al., 2024).

BAG3 is a stress-inducible protein that is barely detectable under physiological conditions in many tissues but is strongly induced by heat shock, proteasome inhibition and oxidative or proteotoxic stress through HSF1 and other stress pathways. Through its IPV and PxxP regions it recruits HSPB8, HSP70 and the ubiquitin ligase CHIP/STUB1, and phosphorylation of its serine residues helps sort clients toward selective autophagy. Early work in normal and neoplastic leukocytes showed that BAG3 modulates stress-induced apoptosis and survival, and hematological malignancies frequently exploit its anti-apoptotic and proteostasis functions (Bonelli et al., 2004; Liu et al., 2022; Arndt et al., 2010).

A related and well-documented feature is the BAG1-to-BAG3 switch in protein quality control. BAG1 preferentially routes HSP70 clients to the ubiquitin-proteasome system, whereas BAG3 recruits the autophagy-lysosomal pathway; during aging and under proteasome impairment the BAG3-to-BAG1 ratio rises and the macroautophagy arm becomes dominant (Gamerdinger et al., 2009). This positions BAG3 as the inducible, autophagy-directed arm of the HSP70 system; whether this switch is engaged in AML has not been tested and is revisited in Sections 3.1, 6.

### 2.3 General autophagy, selective autophagy and CASA: concepts and distinctions

A prerequisite to interpreting the BAG3-HSP70 literature is a precise terminology. Macroautophagy (hereafter autophagy) sequesters cytoplasm in a double-membrane autophagosome and may operate as non-selective bulk degradation or, more commonly, as selective autophagy in which dedicated cargo receptors link specific substrates to LC3/GABARAP. Selective pathways are named for their cargo: CASA targets misfolded and aggregation-prone clients recognized by the HSPB8-HSP70-BAG3-CHIP module; aggrephagy clears protein aggregates through receptors that include SQSTM1/p62, NBR1, TAX1BP1 and OPTN; mitophagy degrades mitochondria through PINK1-PRKN or the receptors BNIP3, BNIP3L/NIX and OPTN. Chaperone-mediated autophagy (CMA) is mechanistically distinct and does not form autophagosomes: HSPA8/HSC70 recognizes proteins bearing a KFERQ-like motif and delivers them to LAMP2A for direct translocation across the lysosomal membrane (Klionsky et al., 2021; Kaushik and Cuervo, 2018; Bauer et al., 2023).

These pathways share the upstream ULK1, PIK3C3/VPS34 and ATG/LC3 machinery but differ in cargo recognition and receptors, whereas CMA shares neither LC3 nor autophagosomes. CASA is best regarded as one specialized, chaperone-defined branch of aggregate clearance rather than a synonym for selective autophagy. Receptors such as SQSTM1/p62 and TAX1BP1 participate in several pathways and are not unique to CASA, and TAX1BP1-directed autophagy can clear protein aggregates independently of BAG3 (Sarraf et al., 2020; Höhfeld et al., 2021).

This terminology carries a logical constraint that runs throughout the review. Demonstrating p62/OPTN-dependent autophagy or mitophagy in AML, or observing LC3-II or p62 changes, does not by itself constitute evidence for BAG3-dependent CASA, because those receptors and that machinery can operate independently of BAG3. A claim of CASA requires evidence that the cargo flux is BAG3-dependent, ideally through BAG3 perturbation (knockdown or knockout) together with a flux readout; evidence for the receptors alone supports selective autophagy, not its BAG3-defined subset (Klionsky et al., 2021; Sarraf et al., 2020).

## 3 Molecular mechanisms of BAG3-HSP70-mediated autophagy in AML

The BAG3-HSP70 axis has been proposed to influence autophagy at initiation, nucleation and maturation, but the strength of evidence differs markedly between systems and between pathways. Throughout Sections 3–5 we therefore classify each claim into four tiers, stated in the text and encoded in Figure 1 and in the tables: Tier A, direct AML evidence (patient samples, AML cell lines or AML genetic models); Tier B, evidence from other hematological settings; Tier C, non-AML evidence (solid tumors, muscle or non-mammalian systems) that remains extrapolative in AML; and Tier D, proposed or hypothetical mechanisms without direct supporting data. This grading allows the strength of support for each pathway to be read directly, and it explicitly separates BAG3-specific evidence from general HSP70 and autophagy findings.

We also distinguish autophagy induction from autophagic flux. An increase in LC3-II or LC3 puncta, or a change in p62 abundance, can reflect either increased autophagosome formation or a block in downstream lysosomal degradation; by itself it does not establish flux. Flux requires evidence of substrate delivery to and degradation in lysosomes, such as LC3-II turnover in the presence of a lysosomal inhibitor, a tandem mRFP-GFP-LC3 reporter, or time-resolved p62 degradation. This distinction is particularly important when interpreting pharmacological agents that themselves impair lysosomes (Klionsky et al., 2021; Yoshii and Mizushima, 2017).

### 3.1 Direct evidence for BAG3 in AML: an inventory

We first inventory the studies that directly manipulate or associate BAG3 in AML and in other hematological malignancies, separating what they establish from what they do not; this is the evidence base against which all mechanistic claims in the review should be measured.

Within AML (Tier A), BAG3 has been linked to anti-apoptotic stability, therapeutic resistance and adverse prognosis rather than to autophagy coupling. In AML cell lines, BAG1 and BAG3 cooperate to maintain BCL2 and MCL1, and BAG3 overexpression rescues the levels of these anti-apoptotic proteins when BAG1 is depleted (Aveic et al., 2011). More recently, elevated BAG3 was identified as a component of a venetoclax-resistance prognostic signature, with BAG3 abundance positively associated with venetoclax IC_50_ (Ke et al., 2023), and a nomogram combining the immune checkpoint CD276, BAG3 and SRC reported that high expression predicts inferior outcome (Wang P. et al., 2023).

Studies in other hematological settings (Tier B) reinforce an anti-apoptotic and proteostasis role. In leukemic cells, BAG3 gene silencing sensitized cells to bortezomib-induced apoptosis, including in a nude-mouse xenograft setting (Liu et al., 2009), and the leukemic oncoprotein WT1 was shown to transcriptionally activate BAG3 (Cesaro et al., 2010). BAG3 is overexpressed and proposed as a therapeutic target in chronic lymphocytic leukemia (CLL) (Zhu et al., 2014), stromal-cell BAG3 shapes the B-CLL microenvironment (Basile et al., 2024), and inhibition of PI3K/AKT signaling suppresses BAG3 and sensitizes diffuse large B-cell lymphoma cells to proteasome inhibitors (Yuan et al., 2019).

This inventory supports a precise conclusion. BAG3 is expressed, prognostically relevant and anti-apoptotic or proteostasis-supportive in AML and other leukemic cells, and its perturbation can modulate sensitivity to bortezomib, venetoclax and proteasome inhibitors. However, none of these studies demonstrates in primary AML that BAG3 directly binds or activates ULK1, that BAG3 or HSP70 competes at the Beclin1 BH3 groove, that HSP70 directly shields ATG5, or that the relevant autophagic flux is BAG3-dependent. The molecular depiction of a BAG3-driven CASA cascade in AML therefore rests on Tier C and Tier D extrapolation rather than on direct AML coupling. This is the central evidence boundary of the review (Figure 1; see also Section 6).

### 3.2 Stage- and context-dependent duality of autophagy in AML

A prerequisite to interpreting BAG3-HSP70 function is to recognize that autophagy is not uniformly protective or suppressive in AML; its net effect depends on disease stage, cellular hierarchy and therapeutic pressure (Figure 1B), and the conditions associated with each net effect are summarized in Table 1.

**TABLE 1:** Context-dependent functions of autophagy in AML and the boundary of BAG3-specific inference.

Disease stage or context	Predominant role of autophagy	Evidence that supports the role	Tier	Does it establish BAG3-dependent CASA?	Primary supports
Early leukemogenesis/pre-MDS	Context-dependent tumor suppression	Atg5/Atg7 loss accelerates MLL–ENL disease; AML blasts show reduced autophagy/lysosome gene expression	A/B	No	Watson et al., 2011, 2015
Established AML/bulk blasts	Predominantly cytoprotective	Atg5/Atg7 deletion depletes leukemia-initiating cells and sensitizes to cytarabine	A	No	Sumitomo et al., 2016
LSC-enriched/hypoxic compartment	Mitophagy and selective autophagy support stemness	AMPK/FIS1, PINK1, OPTN and p62 studies in primary AML, xenografts and AML models	A	No; BAG3 was not perturbed	Pei et al., 2018; Dykstra et al., 2021; Li et al., 2021; Meyer et al., 2023
Therapy-induced adaptation	Protective flux contributes to resistance	JQ1 and FLT3 inhibitors induce protective autophagy; genetic or pharmacological blockade restores sensitivity	A	No	Jang et al., 2017; Xu et al., 2022; Koschade et al., 2022

At the leukemogenic and newly diagnosed stage, autophagy can restrain disease. AML blasts display comparatively low expression of autophagy and lysosomal genes, and genetic or pharmacological inhibition of autophagy increases glycolytic flux and proliferation; heterozygous loss of Atg5 or Atg7 accelerates MLL-ENL-driven leukemogenesis, consistent with a tumor-suppressive brake in early transformation (Watson et al., 2015). Autophagy has likewise been implicated in restraining the pathogenesis of myelodysplastic syndrome (MDS) and early AML (Watson et al., 2011).

In established and LSC-enriched disease, by contrast, autophagy is predominantly cytoprotective. Conditional deletion of Atg5 or Atg7 in a murine myeloid leukemia model cleared leukemia-initiating cells (LICs, the functional leukemia stem cell compartment) and prolonged survival; cytarabine activated autophagy, whereas Atg7 loss sensitized leukemic cells to chemotherapy (Sumitomo et al., 2016). In human AML, AMPK/FIS1-mediated mitophagy is required for primitive-cell self-renewal: FIS1 or AMPK depletion impaired mitophagy, promoted differentiation and reduced serial engraftment, with relative sparing of normal hematopoietic progenitors (Pei et al., 2018). Pharmacological blockade with bafilomycin A1 preferentially targeted hypoxic, stem-like AML blasts by disrupting mitochondrial homeostasis and PINK1-dependent mitophagy (Dykstra et al., 2021); functional dissection identified OPTN as indispensable in AML (Meyer et al., 2023), and a small-molecule inhibitor of the p62 ZZ domain attenuated leukemia-initiation potential (Li et al., 2021). These studies establish LSC-selective mitophagy and receptor-mediated autophagy, but none tested BAG3 dependence.

Therapeutic stress can further shift the balance toward protective autophagy. BET inhibition with JQ1 activates AMPK-ULK1-dependent autophagy in AML stem cells, and blocking this response restores sensitivity (Jang et al., 2017); analogous protective autophagy is induced by FLT3 inhibitors (see Section 4.1). Conversely, selected targeted perturbations can trigger autophagy-dependent cell death, so the outcome is agent- and context-specific (Joffre et al., 2021).

Collectively, these data define a therapeutic window: autophagy inhibition may be most rationally evaluated in established, R/R and LSC-enriched disease, particularly in combination with agents that induce protective autophagy, whereas its use in early or low-burden disease requires caution because of the pathway’s tumor-suppressive and normal hematopoietic stem-cell functions (Saulle et al., 2023; Ianniciello et al., 2018). Whether BAG3-HSP70 is the dominant route of this cytoprotective autophagy in AML, rather than one of several cargo-handling inputs, is a central unresolved question.

### 3.3 Regulation of the ULK1 initiation complex

The ULK1 complex, comprising ULK1, ATG13, FIP200/RB1CC1 and ATG101, is the most upstream kinase assembly of canonical autophagy. It is inhibited by mTORC1 under nutrient-replete conditions and activated by AMPK under energy stress, and ULK1 activity is additionally tuned by post-translational modifications and metabolic cues (Zachari and Ganley, 2017).

Direct AML evidence places ULK1 downstream of oncogenic and therapeutic signaling rather than of BAG3 (Tier A). Oncogenic FLT3-ITD sustains basal autophagy through an ATF4-dependent transcriptional program that is required for blast survival (Heydt et al., 2018), while BET inhibitor JQ1 activates an AMPK-ULK1 autophagic response that protects AML stem cells (Jang et al., 2017). These studies establish ULK1-driven autophagy as functionally relevant in AML without implicating BAG3 as its upstream activator.

A direct physical or functional link between BAG3 and ULK1 has not been demonstrated in AML (Tier D). In glioma cells (Tier C), the BAG3 WW domain was required for autophagy induction, yet BAG3 overexpression in the same study enhanced Bcl-2 binding to Beclin1 and the induced autophagy was Beclin1-independent, arguing against a simple model in which BAG3 directly binds and activates ULK1 (Merabova et al., 2015).

The regulatory direction is not conserved across tissues: in non-small-cell lung cancer cells HSP70 was reported to suppress AMPK activation and autophagy, and combined HSP70 and autophagy inhibition was synergistic (Alhasan et al., 2024), illustrating tissue specificity that cannot be assumed to hold in AML. Determining whether and how the BAG3-HSP70 axis impinges on ULK1 in primary AML cells is a clear experimental priority; until then a BAG3–ULK1 interaction should not be depicted as established.

### 3.4 Modulation of the Beclin1-VPS34 nucleation complex

The Beclin1-VPS34 (PIK3C3) complex, together with ATG14 and other regulators, generates phosphatidylinositol-3-phosphate and initiates phagophore nucleation. Beclin1 contains a BH3 domain whose hydrophobic groove mediates binding to anti-apoptotic Bcl-2 and Bcl-XL; crystal and solution structures of the Bcl-XL-Beclin1 BH3 complex, together with functional analyses, define Beclin1 as a BH3-only protein whose release from Bcl-2/Bcl-XL is governed by BH3-only competitors, phosphorylation and BH3 mimetics (Oberstein et al., 2007; Maiuri et al., 2007; Sinha and Levine, 2008).

There is no convincing structural or biochemical evidence that BAG3 or HSP70 binds the Beclin1 BH3 domain to competitively displace Bcl-2 (Tier D). The BH3 groove recognizes BH3-like helices, whereas the documented BAG3-HSP70 interaction is mediated by the BAG domain and the HSP70 NBD, not by a BH3 interface. Indeed, BAG3 overexpression was found to increase Bcl-2 association with Beclin1 and to support Beclin1-independent autophagy in glioma (Merabova et al., 2015). The principal documented contribution of BAG3-HSP70 to autophagosome formation is instead CASA-mediated cargo delivery, in which p62 oligomers and LC3 engage clients during elongation rather than releasing Beclin1 at nucleation (Tedesco et al., 2023a).

Whether chaperone activity indirectly stabilizes Beclin1 or VPS34 components under proteotoxic stress in AML has not been established; reported effects of BAG3 silencing on Beclin1 derive largely from solid-tumor settings. We therefore frame BAG3-HSP70 modulation of nucleation as an open, indirect possibility rather than a direct competitive mechanism, and we caution against depicting a BAG3-Beclin1-BH3 interaction in AML without supporting data; this boundary is carried into Figure 1 and the conclusions.

### 3.5 Control of autophagosome maturation via ATG proteins and LC3 lipidation

Autophagosome elongation and closure depend on two ubiquitin-like conjugation systems: ATG7/ATG10-mediated ATG12-ATG5-ATG16L1 conjugation and ATG4/ATG7/ATG3-mediated conversion of LC3-I to membrane-bound LC3-II, with p62 and other receptors linking cargo to LC3. LC3-II turnover and p62 degradation are standard readouts of autophagic flux, provided they are interpreted as flux rather than as static abundance (see the Section 3 introduction).

ATG5 is itself a decision point between autophagy and apoptosis. Calpain-mediated truncation of ATG5 generates a pro-apoptotic fragment that translocates to mitochondria and promotes cytochrome c release (Yousefi et al., 2006). A direct physical interaction in which HSP70 binds and shields ATG5 from cleavage has not been demonstrated (Tier D), and the documented cleavage in this switch is calpain-dependent rather than caspase-dependent. The strongest available evidence for HSP70 control of this stage is pharmacological: the HSP70 substrate-binding inhibitor 2-phenylethynesulfonamide (PES, pifithrin-μ) disrupts HSP70 client handling, impairs autophagic flux and compromises lysosome integrity in tumor cells (Leu et al., 2009), and exerts antileukemic and chemosensitizing effects in AML and acute lymphoblastic leukemia models (Kaiser et al., 2011).

The best-supported BAG3-HSP70 contribution at this stage is CASA cargo assembly: the HSPB8-HSP70-BAG3-CHIP complex ubiquitinates and presents clients to p62 and LC3-II for sequestration (Arndt et al., 2010; Tedesco et al., 2023b). Beyond this CASA function, a dedicated role for BAG3 in autophagosome maturation through NF-κB/ATG7 signaling, or a direct HSP70-ATG5 interaction that shields ATG5 from cleavage, is not supported by primary evidence in AML and remains an open question. Functionally, p62-and OPTN-dependent selective autophagy are required for AML LIC maintenance and mitochondrial homeostasis (Meyer et al., 2023; Li et al., 2021), providing an evidence-based link between cargo-handling machinery and the leukemic compartment even where the specific BAG3 connection remains to be proven.

## 4 Context-dependent crosstalk with AML driver mutations

AML is characterized by marked genetic heterogeneity, and recurrent driver mutations reshape metabolism, proteostasis and autophagy. Below we integrate FLT3-ITD, NPM1 and TP53 crosstalk with the BAG3-HSP70 axis, maintaining the four-tier grading and explicitly separating strong autophagy evidence from evidence that specifically implicates BAG3.

### 4.1 FLT3-ITD mutation: autophagy as a basal dependency and a resistance mechanism

FLT3-ITD is among the most common driver mutations in AML and activates proliferative and survival signaling through STAT5, PI3K-AKT-mTOR and RAS-MAPK pathways. Although mTOR signaling is anabolic, FLT3-ITD blasts simultaneously maintain a basal autophagic program required for survival.

Under basal conditions, oncogenic FLT3-ITD signaling induces the transcription factor ATF4, which drives an autophagy/lysosomal gene program; genetic or pharmacological autophagy blockade reduces the viability of FLT3-ITD blasts, identifying autophagy as a non-oncogene addiction in this subtype (Heydt et al., 2018).

Upon treatment with FLT3 inhibitors, leukemic cells mount a protective autophagic response that contributes to resistance. Autophagy inhibition restores sensitivity to FLT3 inhibitors *in vitro* and *in vivo* (Xu et al., 2022), and integrated translatome-proteome profiling independently identified autophagy as an on-target resistance mechanism to FLT3 inhibition (Koschade et al., 2022). Poor clinical response to FLT3 inhibitors has been associated with heightened autophagy and inflammasome signatures in FLT3-ITD patients (Santos de Macedo et al., 2024), combined FLT3 and BTK inhibition was reported to overcome resistance in part by suppressing autophagy (Zhang et al., 2023), and acid ceramidase- and FLT3-ITD-linked autophagy has likewise been implicated in chemoresistance (Zalpoor et al., 2022).

These data establish autophagy as both a basal dependency and an adaptive resistance mechanism in FLT3-ITD AML (Tier A). A specific, non-redundant role for BAG3 within this response has not been directly demonstrated; the closest direct evidence is pharmacological, because covalent HSP70 inhibition degrades FLT3-ITD protein (see Section 5.1). The BAG3-HSP70 axis is therefore a plausible but unproven effector of FLT3 inhibitor-induced protective autophagy, and the two bodies of evidence should not be conflated.

### 4.2 NPM1 mutation: an autophagy dependency independent of demonstrated BAG3 involvement

NPM1 mutations are among the most frequent genetic alterations in adult AML and cause cytoplasmic delocalization of nucleophosmin (NPM1c), perturbing nucleolar integrity, ribosome biogenesis and the ARF/p53 axis and imposing proteotoxic and nucleolar stress (Falini et al., 2009; Colombo et al., 2006).

Direct AML and leukemia-model data show that mutant NPM1 engages core autophagy machinery: NPM1c maintains ULK1 protein abundance through TRAF6-dependent K63-linked ubiquitination, promoting autophagic survival (Tang et al., 2021), and in NPM1-mutated leukemia the glycolytic enzyme PKM2 drives Beclin1-associated autophagic activation to support cell survival (Wang et al., 2019). These findings establish a genuine autophagy dependency in NPM1-mutant AML (Tier A).

Critically, both lines of evidence engage the ULK1 and Beclin1 machinery but currently appear to be independent of demonstrated BAG3 involvement; no BAG3 perturbation is included, and the mechanisms are defined through TRAF6 and PKM2 rather than through the BAG3-HSP70 module. They therefore support an NPM1-linked autophagy dependency but not a BAG3-CASA mechanism, and they should not be cited as evidence that the axis operates in this subtype.

### 4.3 TP53 alterations: reciprocal regulation and a hypothesis-generating extrapolation

TP53 mutations occur in a minority of *de novo* AML but are enriched in therapy-related and complex-karyotype disease, where they confer very poor outcomes and are associated with an adverse proteostasis and differentiation state.

Wild-type p53 regulates autophagy in a dual, compartment-dependent manner, transactivating pro-autophagic genes such as DRAM under stress while suppressing basal autophagy through TIGAR and other effectors. A direct transcriptional link to BAG3 was demonstrated in non-hematopoietic cells, where wild-type p53 represses BAG3 transcription under metabolic stress and BAG3 feeds back to limit p53 accumulation (Wang et al., 2020); this places BAG3 within the p53 stress network (Tier C) but does not establish the relationship in AML.

Mutant p53 is itself a client of chaperone-dependent lysosomal degradation. Chaperone-mediated autophagy, via HSPA8/HSC70 recognition and LAMP2A uptake, degrades mutant p53 (Vakifahmetog et al., 2013), and recent work reports that acetylation promotes mutant TP53-HSPA8 and HSPA8-BAG3 interactions, routing mutant p53 to lysosomes preferentially through CASA (Di Crosta et al., 2025); these latter data derive from solid-tumor models (Tier C) and have not been validated in AML.

On present evidence this section should be read as an emerging and largely hypothesis-generating area. If CASA or CMA contributes to mutant p53 clearance, HSP70 or BAG3 inhibition in TP53-mutant AML could paradoxically stabilize mutant p53 and be detrimental, whereas in wild-type p53 contexts relieving BAG3-mediated feedback might instead augment p53 activity; but the possibility that BAG3/HSP70 inhibition alters mutant p53 stability has not been demonstrated in AML and should not be interpreted as an established genotype-specific therapeutic effect. TP53-stratified preclinical models are needed before clinical translation.

## 5 Therapeutic targeting of the HSP70-BAG3 axis in AML: Preclinical progress

The HSP70-BAG3 axis offers several pharmacologically tractable nodes, but these differ substantially in their target and in their relevance to the specific axis. We therefore separate direct BAG3-HSP70-axis targeting from broader HSP70-family or proteostasis approaches; representative agents are summarized with four evidence dimensions in Table 2 and mapped to their nodes in Figure 1C.

**TABLE 2:** Therapeutic strategies stratified by AML evidence, BAG3 dependence, flux evidence and *in vivo* validation.

Intervention	Direct target and model	Direct AML evidence	BAG3 dependence	Autophagic flux	*In vivo* AML	Tier	Interpretive boundary
VER-155008	ATP-competitive HSP70-family NBD inhibitor; primary AML blasts (Schlecht et al., 2013; Reikvam et al., 2013)	Yes: antiproliferative and pro-apoptotic; additive with 17-DMAG	No	No	No	A	Broad HSP70-family agent
PES/pifithrin-μ	HSP70 SBD ligand with lysosomal effects; acute leukemia lines and primary AML (Leu et al., 2009; Kaiser et al., 2011; Yang et al., 2021)	Yes: IC50 values of approximately 2.5–12.7 μM in lines and approximately 8.9 μM in primary blasts	No	Yes, but mixed chaperone/lysosomal perturbation	No	A	Flux effect is not autophagy-specific
QL47	Covalent HSP70 inhibitor at Cys267; FLT3-ITD AML (Hu et al., 2021)	Yes: FLT3-ITD degradation, apoptosis and activity against TKD-resistant cells	No	No	Yes	A	76.64% xenograft growth inhibition at 10 mg/kg/day
VER-155008 + 17-DMAG	Dual HSP70/HSP90 network inhibition; primary AML (Reikvam et al., 2013)	Yes: additive antileukemic activity	No	No	No	A	Proteostasis-network intervention
JG-98	Allosteric HSP70–BAG3 PPI inhibitor; non-AML cancer models (Li et al., 2015; Yaglom et al., 2018)	No	Direct PPI inhibition; downstream responses can be BAG3-dependent or independent	No	No; solid-tumor xenografts only	C	No AML genetic validation
AZD7762-induced BAG3 loss	Reported CRBN-dependent BAG3 degradation; non-AML models (Liao et al., 2024)	No	BAG3 abundance decreases; selective degrader pharmacology is not established	No	No	C	Pleiotropic kinase inhibitor
YUM70	GRP78/HSPA5 inhibitor; pancreatic cancer (Samanta et al., 2021)	No	No	No	No	C	ER paralog, outside cytosolic axis
Hsc70-recruiting PROTAC	Uses HSP70/Hsc70 machinery to degrade GPX4 or BRD4; non-AML models (Dong et al., 2026)	No	No	No	No	C	Not an HSP70 degrader

### 5.1 HSP70 inhibitors: mechanisms and preclinical evidence

#### 5.1.1 VER-155008

VER-155008 is an adenosine-derived, ATP-competitive inhibitor that binds the HSP70 NBD. It is not strictly isoform selective, and functional profiling indicates that effective blockade can require concurrent suppression of constitutive and inducible HSP70 paralogs (Schlecht et al., 2013). In primary human AML blasts, VER-155008 inhibited proliferation and induced apoptosis, and its combination with the HSP90 inhibitor 17-DMAG produced additive antileukemic effects without the compensatory heat-shock induction seen with HSP90 inhibition alone (Reikvam et al., 2013). VER-155008 remains preclinical for hematological malignancies, with pharmacokinetic and selectivity limitations that have restricted *in vivo* and clinical development; it targets the HSP70 family rather than the BAG3 interaction.

#### 5.1.2 PES/pifithrin-μ

2-Phenylethynesulfonamide (PES, pifithrin-μ) targets the HSP70 substrate-binding domain—recent work shows it covalently modifies Cys574 and Cys603 of inducible HSP70—and, importantly, also disrupts lysosome integrity and autophagic flux, coupling chaperone inhibition to lysosomal dysfunction (Leu et al., 2009; Yang et al., 2021). In AML and acute lymphoblastic leukemia cell lines, PES showed IC50 values of approximately 2.5–12.7 μM, with a median IC50 of about 8.9 μM in primary AML blasts, and relative sparing of normal hematopoietic cells; it sensitized leukemic cells to cytarabine, 17-AAG, vorinostat and sorafenib (Kaiser et al., 2011). Its mixed chaperone/lysosomal mechanism, which is not autophagy-specific, should be considered when interpreting flux-based conclusions.

#### 5.1.3 QL47

QL47 is a covalent HSP70 inhibitor that reacts with Cys267 within a large groove of the NBD near, but not occupying, the ATP pocket, and it does not inhibit the HSP70 ATPase; it promotes degradation of client proteins. In FLT3-ITD-positive AML it triggers degradation of FLT3-ITD itself and overcomes resistance conferred by FLT3 tyrosine-kinase-domain mutations, and in patient-derived FLT3-ITD cells it induced apoptosis. Importantly, antileukemic efficacy was demonstrated *in vivo*: in an MV-4-11 bone-marrow engraftment model QL47 extended survival and reduced marrow leukemic burden, and in a subcutaneous MV-4-11 xenograft it achieved 76.64% tumor growth inhibition at 10 mg/kg/day (Hu et al., 2021). Because QL47 also inhibits eukaryotic translation, its antitumor activity may reflect dual HSP70 and translation effects, and it is not a BAG3-directed agent.

#### 5.1.4 YUM70

YUM70 is a hydroxyquinoline inhibitor of the ER-resident HSP70 paralog GRP78/HSPA5, which induces ER-stress-mediated apoptosis and disrupts GRP78-mediated client handling in pancreatic cancer (Samanta et al., 2021). It must be emphasized that YUM70 is not a pan-HSP70 inhibitor and does not directly target cytosolic HSP70 or BAG3; no AML data are currently available. Its inclusion is to delineate, rather than conflate, HSP70-family targets, and any AML application remains extrapolative.

### 5.2 Beyond ATP-site inhibition: PPI blockade, dual chaperone targeting, BAG3-directed agents, degradation and delivery

Across the compounds discussed below, a recurring limitation is poor drug-likeness. Most were identified more than a decade ago, are active at micromolar concentrations *in vitro*, show limited paralog or pathway selectivity, and have little or no AML *in vivo* or clinical validation. This explains why renewed medicinal-chemistry effort is focused on protein-protein interaction inhibitors, BAG3-directed agents and targeted degraders rather than on further optimization of the early ATP-site compounds.

Targeting the HSP70–BAG3 interaction rather than the HSP70 ATP site offers the most direct axis-selective route. JG-98 is an allosteric HSP70 modulator that disrupts HSP70–BAG3 binding, with EC_50_ values of roughly 0.3–4 μM across non-AML cancer cell lines, and destabilizes clients such as FoxM1 (Li et al., 2015). However, pathway profiling showed that some JG-98 responses are BAG3-dependent whereas others, including changes in AKT and MYC, are BAG3-independent (Yaglom et al., 2018). JG-98 has not been evaluated in AML. It therefore supports the interaction as a pharmacologically tractable node in non-AML models but does not establish that its antitumor activity is uniformly BAG3-mediated or that AML is dependent on this interaction.

Dual chaperone targeting is mechanistically attractive because HSP70 and HSP90 cooperate in client maturation. Simultaneous inhibition of constitutive HSC70 and inducible HSP72 was required to fully disable HSP90-dependent client folding and induce tumor-specific apoptosis (Powers et al., 2008), and comparative profiling shows HSP70 inhibitors also suppress HSP90 clients and autophagy (Budina-Kolomets et al., 2014). In primary AML, combining VER-155008 with the HSP90 inhibitor 17-DMAG was additive (Reikvam et al., 2013), supporting network-level rather than single-paralog targeting; this is a proteostasis-network rather than a BAG3-specific strategy. Small molecules that trigger CRBN-dependent BAG3 degradation have also been described in non-AML settings (Liao et al., 2024).

Direct BAG3-directed strategies, including antibodies against extracellular or secreted BAG3 and antisense or oligonucleotide approaches, are at an early stage and must contend with the pleiotropy and compartment-specific functions of the protein (Kirk et al., 2021). AZD7762 has been reported to induce CRBN-dependent BAG3 degradation in non-AML models, but it is a pleiotropic kinase inhibitor and has not established a selective BAG3-degrader mechanism or AML efficacy (Liao et al., 2024). Targeted protein degradation remains relevant as a platform, although its terminology must be precise: the reported HSP70-interactome-mediated PROTAC recruits Hsc70/HSP70 machinery to degrade proteins such as GPX4 or BRD4; it does not degrade HSP70 itself (Dong et al., 2026). Broader degrader strategies are advancing in hematological malignancies, but neither selective BAG3 degradation nor HSP70–BAG3-axis degradation has been validated in AML (Feng et al., 2024).

Delivery innovation may help overcome selectivity and toxicity barriers. A carrier-free, peptide-directed nanoassembly co-delivering daunorubicin and an siRNA against the AML target LILRB4 showed targeted activity in THP-1 cells and an AML mouse model (Yang et al., 2025), illustrating a platform that could in principle carry BAG3/HSP70-directed cargo to leukemic cells while limiting cardiac exposure; application to the axis itself remains hypothetical.

### 5.3 Combination therapeutic strategies: evidence-supported versus proposed

Rational combinations may outperform single-agent chaperone targeting, but evidence-supported combinations must be separated from proposed future ones.

Evidence-supported combinations with chemotherapy include the following: PES enhanced the activity of cytarabine, 17-AAG, vorinostat and sorafenib in acute leukemia (Kaiser et al., 2011); VER-155008 plus 17-DMAG was additive in primary AML (Reikvam et al., 2013); and recent work reports that targeting LAPTM5 enhances AML sensitivity to cytarabine through inhibition of autophagy (Zeng et al., 2026). With FLT3 inhibitors, autophagy or HSP70 blockade restores sensitivity *in vitro* and *in vivo*, and covalent HSP70 inhibition additionally degrades FLT3-ITD (Xu et al., 2022; Koschade et al., 2022; Zhang et al., 2023; Hu et al., 2021).

In contrast, combining chaperone or autophagy blockade with azacitidine-venetoclax regimens is mechanistically logical—given the proteostasis and BAG3 associations with venetoclax resistance—but is, to our knowledge, untested; we identify it explicitly as a research hypothesis rather than an evidence-supported regimen. Proposed extensions with immune checkpoint or macrophage-checkpoint blockade likewise require direct AML testing (see Sections 5.4 and 6).

### 5.4 Immunotherapy: an emerging and currently indirect direction

The proposed connection between BAG3-HSP70/autophagy targeting and immunotherapy is interesting but currently indirect; because part of the rationale derives from non-AML studies, we present it as an emerging research direction rather than an established therapeutic strategy.

Secreted or extracellular BAG3 has documented non-AML signaling roles. In pancreatic ductal adenocarcinoma, tumor-derived BAG3 activates macrophages through IFITM2 to support tumor growth (Rosati et al., 2015); extracellular BAG3 participates in vascular homeostasis (Carrizzo et al., 2016); and BAG3-associated macrophage programs have been defined in other solid tumors (Zhang et al., 2026). These support anti-BAG3 or macrophage-directed approaches but provide no direct AML evidence.

Cell-surface chaperones are being explored as immune effectors: CAR-NK cells that target membrane HSP70 are in preclinical development (Hachani et al., 2020), and chronic ER stress can expose cell-surface chaperones as candidate CAR targets in AML, although the latter remains at a discovery stage. Autophagy is also coupled to MHC-I antigen presentation in solid-tumor models, where autophagic flux and ATG7 correlate with MHC-I/HLA-E modulation (Luo et al., 2026), and AML LSCs use distinct sialylglycan-Siglec evasion mechanisms (Peng et al., 2026). A specific, BAG3-dependent AML component of these immunological links is not established.

### 5.5 Translational challenges: therapeutic window, cardiac safety and biomarkers

The therapeutic window between AML/LSCs and normal hematopoietic stem cells deserves particular emphasis because autophagy has physiological functions in normal hematopoiesis. Atg7 is essential for normal HSC maintenance (Mortensen et al., 2011), FOXO3A directs a protective autophagy program in hematopoietic stem cells under stress (Warr et al., 2013), and notably normal HSCs preferentially traffic misfolded proteins through aggresomes and aggrephagy rather than CASA (Chua et al., 2023). The distinct proteostasis strategy of normal HSCs offers a possible basis for selectivity, but it is not yet evidence that BAG3-CASA inhibition will spare normal cells, and the window must be measured directly.

BAG3 also has essential functions in cardiomyocytes, where it maintains proteostasis, mitochondrial function and inflammasome regulation, and BAG3 deficiency or mutation causes cardiomyopathy and heart failure (Kirk et al., 2021; Wang J. et al., 2023; Knezevic et al., 2015); BAG3 additionally regulates mitophagy and TGF-beta receptor signaling in cardiac fibroblasts (Martin et al., 2024; Wang et al., 2025). Systemic BAG3 targeting therefore carries a cardiac-safety liability that may require cardiac-sparing or leukemic-directed delivery.

Patient selection and biomarker development are further priorities. HSP70 overexpression and circulating HSP70 have been associated with adverse features and tumor burden in AML (Thomas et al., 2005; Yeh et al., 2010), and autophagy-gene and immune signatures have shown prognostic value (Zhu et al., 2024). Prospective biomarkers should include basal and drug-induced autophagic flux measured as flux (LC3-II and p62 turnover with a lysosomal inhibitor), HSF1 target induction, BAG3 and HSP70 paralog expression, the LSC/minimal residual disease fraction, and FLT3/NPM1/TP53 genotype. No HSP70 or BAG3 inhibitor has yet entered AML clinical practice, and trials should be designed around biomarker-enriched, R/R and LSC-high populations.

## 6 Current knowledge gaps and future research priorities

Drawing the preceding evidence together, the major unresolved questions can be grouped as follows; the first three define the central mechanistic boundary identified in this review. The gaps and the experiments that would resolve them are compiled in Table 3.

1. Is BAG3 a functional dependency in AML? The inventory in Section 3.1 establishes expression, prognostic relevance and anti-apoptotic or proteostasis roles, but not a non-redundant dependency. This requires CRISPR-based BAG3 knockout or inducible knockdown in patient-derived AML and patient-derived xenograft models, together with rescue, rather than additional correlative analyses.
2. Does BAG3 directly regulate AML autophagic flux? CASA flux—HSPB8-HSP70-BAG3-CHIP-p62 client delivery to lysosomes—should be measured as flux in primary AML using tandem reporters and lysosomal turnover, with BAG3 perturbation to establish that the flux is BAG3-dependent.
3. Does BAG3-dependent CASA contribute to LSC maintenance? This should be tested in LSC-enriched and hypoxic compartments where OPTN- and p62-dependent mitophagy is already established, separating any BAG3 contribution from the receptor-mediated autophagy that is known to operate.
4. Are the three proposed mechanistic links real? Direct BAG3–ULK1 coupling, BAG3/HSP70 competition at the Beclin1 BH3 groove, and HSP70 shielding of ATG5 from cleavage should either be demonstrated with structural, biochemical and genetic evidence in AML or explicitly retired from the mechanistic model and the figure.
5. How do these effects vary by FLT3, NPM1 and TP53 genotype? NPM1-linked autophagy appears independent of demonstrated BAG3 involvement, a non-redundant BAG3 role in FLT3-ITD disease is unproven, and the possibility that chaperone inhibition stabilizes mutant p53 must be tested before any genotype-specific use.
6. Can an adequate therapeutic window and cardiac safety be achieved? Selectivity relative to normal hematopoietic stem cells—which favor aggresomes and aggrephagy rather than CASA—and cardiac-sparing or leukemic-directed delivery, together with flux- and LSC-based pharmacodynamic biomarkers, should define the translational path; the immunotherapeutic rationale requires direct AML rather than non-AML evidence.

**TABLE 3:** Experimental roadmap for resolving the principal knowledge gaps.

Knowledge gap	Recommended experiment/Model	Decisive readout
Is BAG3 a functional dependency?	Inducible CRISPR knockout or knockdown of BAG3 in patient-derived AML and PDX models, with BAG3 re-expression rescue	Loss of viability or engraftment that is rescued; distinction from BAG1 compensation
Does BAG3 directly regulate AML autophagic flux?	Tandem mRFP-GFP-LC3 and LC3-II/p62 turnover with and without a lysosomal inhibitor, combined with BAG3 perturbation	Reduced BAG3-dependent cargo delivery and lysosomal turnover, not merely LC3-II accumulation
Does BAG3-CASA contribute to LSC maintenance?	BAG3 perturbation in sorted LSC and hypoxic fractions; serial colony-forming and xenograft repopulation assays	Reduced self-renewal and serial engraftment attributable to BAG3, separated from OPTN/p62 pathways
Are the three proposed links real?	Co-immunoprecipitation, structural assays and epistasis for BAG3–ULK1, Beclin1 BH3 competition and HSP70–ATG5	Positive biochemical or genetic evidence, or explicit removal of the links from model and figure
How do effects vary by FLT3, NPM1 and TP53 genotype?	Isogenic and patient-derived models stratified by genotype, with mutant-p53 stability readouts	Genotype-specific BAG3 dependence; test whether chaperone inhibition stabilizes mutant p53
Can an adequate therapeutic window and cardiac safety be achieved?	Normal HSC co-culture, cardiomyocyte and fibroblast assays, cardiac-sparing delivery; pharmacodynamic biomarkers	Spared normal HSC and cardiac function; flux-, LSC- and BAG3/HSP70-based pharmacodynamic markers

## 7 Discussion and conclusion

The BAG3-HSP70 axis is a stress-responsive proteostasis node whose best-defined autophagic function in canonical non-AML systems is CASA-mediated cargo delivery through HSPB8-HSP70-BAG3-CHIP-p62-LC3. A critical reading under the four-tier framework gives a clear result: direct AML evidence is comparatively strong for the cytoprotective, mitophagic and chemoresistance roles of autophagy and HSP70 in established and therapy-stressed LSCs, and for FLT3-ITD- and NPM1-linked autophagy, but it does not establish a specific BAG3 dependency. We therefore state as a principal conclusion that direct BAG3 coupling to ULK1, competitive BAG3/HSP70 binding at the Beclin1 BH3 domain, and direct HSP70 shielding of ATG5 from cleavage are unsupported and are more accurately framed as untested or tissue-specific hypotheses (Tier D); NPM1-linked autophagy is presently independent of demonstrated BAG3 involvement, and the TP53 connection is hypothesis-generating (Figure 1).

The therapeutic toolkit has broadened beyond ATP-site HSP70 inhibitors to include covalent and substrate-binding compounds, the HSP70-BAG3 protein-protein interaction inhibitor JG-98, dual HSP70/HSP90 network inhibition, BAG3-directed agents, degraders and targeted nanoassemblies. However, only the broad HSP70-family agents—most notably QL47, which carries AML *in vivo* evidence—are supported directly in AML, whereas the most direct axis-targeting agent (JG-98) remains untested in AML (Table 2); grouping these without distinguishing their target and evidence level would overstate direct axis-targeting.

Three priorities should guide future work. First, the physical and functional coupling of BAG3 to the ULK1 and Beclin1 complexes, and the BAG3 dependence of autophagic flux, must be tested directly in primary AML cells and AML models rather than inferred from solid tumors or muscle. Second, BAG3 dependence should be mapped across disease stage and FLT3-ITD, NPM1 and TP53 genotype, including the possibility that chaperone inhibition stabilizes mutant p53. Third, the ability of HSP70/BAG3 or autophagy inhibition to synergize with FLT3 inhibitors, azacitidine-venetoclax (currently a hypothesis) and immune or macrophage checkpoint blockade through the proposed restoration of MHC-I visibility and clearance of LSCs should be evaluated alongside normal-stem-cell and cardiac-safety endpoints. Addressing these questions will determine whether the BAG3-HSP70 axis can be converted from a plausible mechanism into a precise, biomarker-driven therapeutic target in AML.
